# Sublethal Toxicity and Gene Expression Changes in *Hydra vulgaris* Exposed to Polyethylene and Polypropylene Nanoparticles

**DOI:** 10.3390/nano15130954

**Published:** 2025-06-20

**Authors:** Joelle Auclair, Chantale André, François Gagné

**Affiliations:** Environment and Climate Change Canada, Aquatic Contaminants Research Division, Montréal, QC H2Y 2E7, Canada; joelle.auclair@ec.gc.ca (J.A.); chantale.andre@ec.gc.ca (C.A.)

**Keywords:** *Hydra vulgaris*, sublethal toxicity, oxidative stress, regeneration, DNA repair

## Abstract

Plastic nanoparticles (NPs) released from plastic breakdown pervade aquatic ecosystems, raising concerns about their long-term toxic effects in aquatic organisms. The purpose of this study was to examine the sublethal toxicity of polyethylene (PeNPs) and polypropylene (PpNPs) nanoparticles of the same size (50 nm diameter) in *Hydra vulgaris*. Hydras were exposed to increasing concentrations of PeNPs and PpNPs (0.3–10 mg/L) for 96 h at 20 °C. Toxicity was determined based on the characteristic morphological changes and gene expression analysis of genes involved in oxidative stress, DNA repair, protein salvaging and autophagy, neural activity and regeneration. The data revealed that PpNPs produced morphological changes (50% effects concentration EC50 = 7 mg/L), while PeNPs did not. Exposure to both nanoplastics produced changes in gene expression in all gene targets and at concentrations less than 0.3 mg/L in some cases. PpNPs generally produced stronger effects than PeNPs. The mode of action of these plastic polymers differed based on the intensity of responses in oxidative stress (superoxide dismutase, catalase), DNA repair of oxidized DNA, regeneration and circadian rhythms. In conclusion, both plastics’ nanoparticles produced effects at concentrations well below the appearance of morphological changes and at concentrations found in highly contaminated environments.

## 1. Introduction

Plastic products pervade most compartments in the environment (soil, sediment and surface waters) due to massive production and inefficient recycling and waste disposal activities [1,2]. Plastics are generally perceived as inert, safe and inexpensive materials resulting in a wide range of applications (cars, furniture, households) and an inevitable material for food containers and clothing. The inadequate disposal management of these plastic materials contributes to their release in the environment, finding their way into both freshwater and marine ecosystems worldwide. It is estimated that only 9% of plastic materials are recycled, with 19% destined for incineration and the remainder in landfills [3]. In addition, it was estimated that 20–25% of plastic waste ends up in uncontrolled dumpsites and is ultimately released in adjacent terrestrial and aquatic environments. Polypropylene (Pp) is used for face masks, which were massively used during the COVID-19 pandemic and significantly contributed to the already growing plastic contamination problem [4,5]. Pp and polyethylene (Pe), along with polystyrene and polyvinyl, represent the most common plastic materials, and they are often detected in various ecosystems [6,7]. The weathering and degradation of plastics are relatively slow, leading to the gradual breakdown of plastic debris into microparticles (<5 mm–1 µm) and nanoparticles (1–1000 nm). Nanoplastics (NPs) are considered more dangerous to organisms since they can easily pass through biological barriers, finding their way into various organs and being absorbed in cells by various mechanisms such as phagocytosis and pinocytosis [8]. The reported levels of nanoplastics in wastewaters and rivers from largely populated areas were between 0.1 and 100 µg/L [9,10]. Maximum concentrations reached the upper µg/L range (500–1000 µg/L) in some cases. It appears that most plastic nanoparticles were from domestic plastic water emissions and street runoffs among urban sites, while in rural sites, the contribution results from aquaculture, agriculture and road runoffs from tire wear materials. The aquatic toxicity was mostly examined in fish [11,12]. These studies reveal that microplastic contamination is much better understood than contamination by nanoparticles, although they can transport existing chemicals such as pharmaceuticals, additives and metals. Some ecotoxicity studies on invertebrates, such as bivalves, nematodes and crustaceans (daphnids), could be found, which also revealed deleterious effects from oxidative stress [13,14,15]. However, few studies exist on the toxicity, at both organismal and molecular levels, in small-sized freshwater invertebrates. The cnidarian *Hydra vulgaris* Pallas, 1766 is a sensitive, small-sized (1–4 mm) invertebrate of the benthic community that can be used for toxicity assessments of miscellaneous chemicals and liquid mixtures [16,17]. Hydra is commonly found in tropical and temperate freshwaters attached to various substrates such as rocks, tree barks and plants. It is a relatively simple organism composed of two layers of cells forming a head with five to seven tentacles attached to a tubular body (5–10 mm long), itself attached to a solid substrate (Figure S1). The head comprises the tentacles and the mouth for food intake and exudation of detritus and nondigested material from the tubular body. The tubular body is composed of two parts: the mouth and digestive system at the upper end and the lower body for reproduction, where polyp offspring emerge from the body. Hydras feed on small invertebrates such as copepods and water fleas. They are solitary and sessile organisms and reproduce asexually most of the time. These organisms have remarkable regenerative abilities, growing and reproducing without significant aging. Reproduction occurs through a budding process at the lower part of the tubular body (Figure S1), with a population doubling time of 4–5 days, depending on the experimental conditions such as temperature, media composition and feeding rates [17]). One attractive feature of the hydra is that it resides in its sensitivity towards contaminants, often better than salmonids, and is considered a viable alternative to fish bioassays. There is a pressing need to reduce/replace fish for the toxicity assessments needed for compliance surveillance and monitoring of complex effluents and leachates in the environment. Toxicity is usually measured by a series of morphological changes following the severity of toxic effects. Reversible (sub-lethal) morphological changes consist of antennae shortening (at least more than one third their original length) with budding at the tip of the antennae. If toxicity reaches lethal levels, severe antennae and body compression forming a tulip-like appearance is observed and indicates irreversible (death) damage. Because of the small size of hydra, biomarkers assessment studies are wanting at the present to understand changes at the molecular level that precede morphological changes. A quantitative polymerase chain reaction methodology has been recently developed to understand the sublethal effects of various emerging contaminants before the onset of morphological changes [18]. This toxicogenomic platform involves gene expression for oxidative stress (superoxide dismutase, catalase), DNA repair (8-oxoguanine excision), damaged protein recycling (ubiquitination and autophagy), neural activity (dopamine decarboxylase and neuronal cell repair) and differentiation (under circadian rhythm). Plastic NPs are well recognized for inducing oxidative stress and increasing protein ubiquitination in exposed organisms, leading to lipid peroxidation, DNA damage and mechanisms for the removal of denatured proteins [8,19]. NPs have been shown to induce protein denaturation, leading to ubiquitin tagging for removal by the proteasome–ubiquitin pathway in cells [20]. The above studies revealed that important changes in gene expression are at play before the onset of morphological changes in hydra. The sublethal effects of plastic nanoparticles are not well understood in aquatic invertebrates and merit investigation for the protection of the aquatic benthic community.

The purpose of this study was, therefore, to examine the sublethal toxicity of PeNPs and PpNPs to *Hydra vulgaris*. The Pe and PpNPs were of the same size range (50–70 nm) to minimize size-related effects. Given that Pe and Pp are non-polar aliphatic polymers, their toxic properties should be similar for NPs of similar sizes at the nanoscale (null hypothesis) provided that they contain similar amounts of plasticizers or other additives. The toxic properties were examined at the morphological changes (shortening of tentacles and budding) and at the gene-expression levels from the physiological targets described above. An attempt was made to relate gene expression changes with toxicity effects based on morphological observations.

## 2. Materials and Methods

### 2.1. Reagents and Nanoplastic Preparation

The plastic nanoparticles (NPs) were purchased from CD Bioparticles (Shirley, NY, USA). Diagpoly^TM^ polyethylene nanoparticles (PeNPs) with a diameter of 50 nm (polydispersity index <0.1) were purchased as 1% concentration (10 mg/mL) in the suspension media composed of 0.1% Tween-20 and 2 mM NaN_3_. This suspension contained 1 × 10^14^ NPs/mL. DiagPoly™ Polypropylene nanoparticles (PpNPs), 50 nm (PDI < 0.2), supplied at 10 mg/mL in the same suspension media and contained the same number of NPs as with PeNPs. The vehicle thus contained Tween 20 and NaN_3_, from which is considered the solvent control (vehicle). The highest exposure concentration of 10 mg/L therefore contained 0.0001% Tween 20 and 2 µM NaN_3_ and constituted the solvent control. No sublethal and lethal morphological changes were observed for the vehicle. Preliminary experiments were performed to evaluate the solubility based on turbidity measurements at 600 nm using a microplate reader (Synergy IV, Biotek instrument, Winooski, VT, USA). A 100 mg/L and 10 mg/L solution was prepared in the hydra exposure media and allowed to stand for 1 and 96h at room temperature in the absence of hydra. Then, 200 µL of the suspension was collected in clear microplates and measured at 600 nm.

### 2.2. Aquatic Toxicity Assessment with Hydra Vulgaris

Exposures of *Hydra vulgaris* to Pp and PeNPs followed a standard methodology, as described elsewhere [21,22]. The organisms were grown in 100 mL of the hydra medium: 1 mM CaCl_2_ containing 0.4 mM TES buffer pH 7.5. They were fed every second day to brine shrimps (*Artemia salina*) after carefully rinsing them in hydra medium to remove excess salt. Hydras were not fed 12h prior to the initiation of the exposure experiments. Three individuals (n = 3) were added in each well (3 wells) with 4 mL of the hydra medium in 12-well clear microplates. Hydras were treated to concentrations of PeNPs and PpNPs (0, 0.3, 0.6, 1.25, 2.5, 5 and 10 mg/L) for 96 h at 20 °C. The exposure concentrations corresponded to 3, 6, 12.5, 25, 50, 100 × 10^9^ particles/mL. The selected concentrations were based on a range finding for the sublethal/lethal toxicity, as recommended by the standard protocol [22] (Environment and Climate Change Canada, 2020). These concentrations represent the upper range of plastics in the wastewaters [10]. Two other microplates were prepared as described above to ensure enough hydras for gene expression analysis. Sublethal and lethal toxicity were determined by characteristic morphological changes Figure S1) observed using a 4–6× stereomicroscope, where sublethal effects were observed by budding and shortening of tentacles and lethal/irreversible effects by severe tentacle retraction leading to tulip-like body. The lethal concentration that kills 50% of the hydras (LC50) and the sublethal effect concentration of 50% of the hydras (EC50) were determined using the Spearman–Karber method [23]. Morphologically unaffected hydras were collected for gene expression analysis. Hydras were harvested with a 1 mL pipet, immediately transferred in RNA later solution (Millipore Sigma, Oakville, ON, Canada) and stored at −20 °C for gene expression analysis.

### 2.3. Gene Expression Analysis

Gene expression analysis was determined by a newly developed quantitative reverse transcriptase polymerase chain reaction (qRTPCR) for *Hydra vulgaris* (Auclair et al., 2024) [18]. A total of 9 hydras (3 wells) was sufficient for RNA extraction by solid-phase electrophoresis (Agilent RNA ScreenTape Assay (cat # 5067-5576, Agilent Technologies Inc., Santa Clara, CA, USA). Quantitative RT-PCR analysis was conducted following the reported levels of gene targets from Auclair et al. (2024) [18] and are described (Table 1). HPRT, RPLPO, Efα were selected as possible reference gene for normalization with baseline RNA levels. The data were expressed as relative mRNA levels relative to controls. Gene expression between media and solvent controls were generally unchanged, with the exception of the following gene transcripts showing a small (<1.2-fold) but significant decrease in the solvent controls: RPlPO, MAPC3, MANF1 and OGG.

### 2.4. Data Analysis

The exposure experiments were repeated twice, with three microplates comprising 3 hydras per well and 3 replicate wells per exposure concentration. The data were expressed the effect threshold: (no effect concentration x lowest significant effect concentration)^1/2^ in mg/L. The gene expression data were analyzed using a rank-based analysis of variance followed by the Conover–Iman test for differences from the controls. Relationships between gene expression data were determined using the Pearson moment procedure. The gene expression data were also analyzed by discriminant function analysis to seek out similarities (or differences) between the effects from PeNPs and PpNPs and determine the genes whose expressions best discriminate between PpNPs and PeNPs. Significance was set at *p* < 0.05. All the statistical analyses were conducted using SYSTAT (version 13, Palo Alto, CA, USA).

## 3. Results

**Figure 1:**
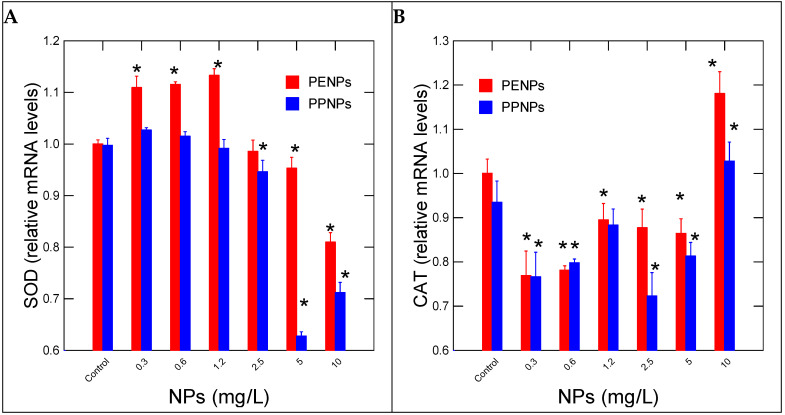
Oxidative stress in hydras exposed to PE and Pp nanoplastics. Relative levels of SOD (**A**) and CAT (**B**) mRNA levels were evaluated in hydras exposed to NPs for 96 h. The data represents the mean with the standard levels. The star (*) symbol represents significance at α < 0.05 levels from the control (vehicle only) and highlights the threshold concentrations.

No evidence of NP loss was observed based on turbidity measurements in the incubation and precipitation. Size analysis revealed that the mean diameters of the NPs were similar (50 nm), with a polydispersity index of 0.2 in the stock solutions. The reported Zeta potential was between −10 mvolt [24], hence the presence of tween-20 detergent in the suspension and its inclusion as the solvent control. Based on turbidity measurement, no changes were observed between 1 and 96 h dissolution in the hydra medium (containing 1 mM CaCl_2_ and 0.1 mM MOPs buffer). The toxicity of Pe and Pe NPs in hydras revealed no lethal effects for concentrations up to 10 mg/L. Sublethal effects were observed only for PpNPs with an EC50 = 7 mg/L. The effects of PeNPs and PNPs on oxidative stress were examined by CAT and SOD gene expression (Figure 1). For PeNPs, an increase in SOD activity was observed at 0.3–1.25 mg/L, with a gradual decrease at higher concentrations (Figure 1A). For PpNPs, decreased SOD gene expression occurred at a threshold concentration of 1.8 mg/L, which is below the EC50 value of 7 mg/L. The expression of CAT gene expression followed a biphasic response to both PeNPs and PpNPs (Figure 1B). CAT gene expression was decreased at low concentrations between 0.3 and 5 mg/L, followed by an increase at 10 mg/L. SOD and CAT were negatively correlated only for PeNPs (−0.71) (Table 2).

**Figure 2:**
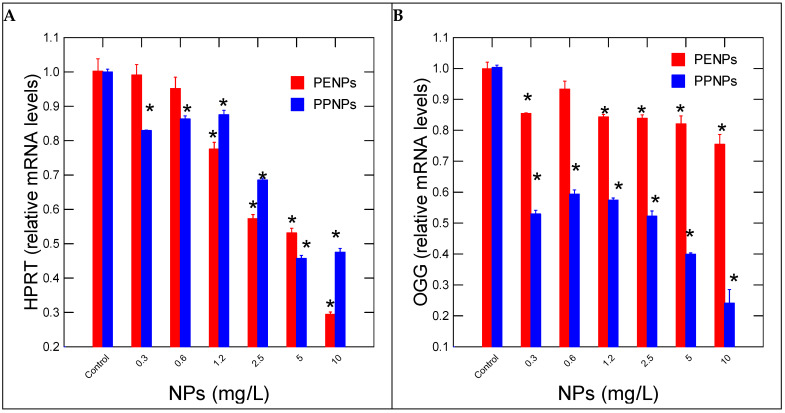
DNA synthesis and repair activity in hydras exposed to plastic nanoparticles. Relative levels of HPRT (**A**) and OGG DNA repair (**B**) mRNA levels were measured in hydras exposed to NPs for 96h. The data represents the mean with the standard levels. The star (*) symbol represents significance at α < 0.05 levels from the control (vehicle only) and highlights the threshold concentrations.

Changes in the purine salvage pathway (HPRT) and repair (8-oxo-guanosine) were determined in hydras exposed to plastic NPs (Figure 2). For PeNPs, the gene responsible for recycling guanine and inosine in the purine salvage pathway (HPRT) was significantly reduced at a threshold concentration of 0.88 mg/L For PpNPs, and the purine salvage pathway was significantly reduced at the lowest concentration of 0.3 mg/L. This suggests that in both cases, DNA turnover was decreased upon exposure to NPs. For PeNPs, correlation analysis revealed that HPRT gene expression was correlated with CAT (r = −0.43) and SOD (r = 0.72). For PpNPs, correlation analysis revealed that HPRT gene expression was correlated with SOD (r = 0.89). In respect to OGG gene expression involved in the repair of 8-oxoguanosine adducts, the gene expression was decreased at the lowest exposure concentration for both NPs (Figure 2B). The intensity of inhibition was stronger for PpNPs than PeNPs, with 2- and 1.2-fold inhibitions, respectively. Correlation analysis revealed that OGG gene expression was correlated with HPRT gene expression (r = 0.72) for PeNPs. For PpNPs, correlation analysis revealed that OGG gene expression was correlated with SOD (r = 0.63) and HPRT (r = 0.87).

**Figure 3:**
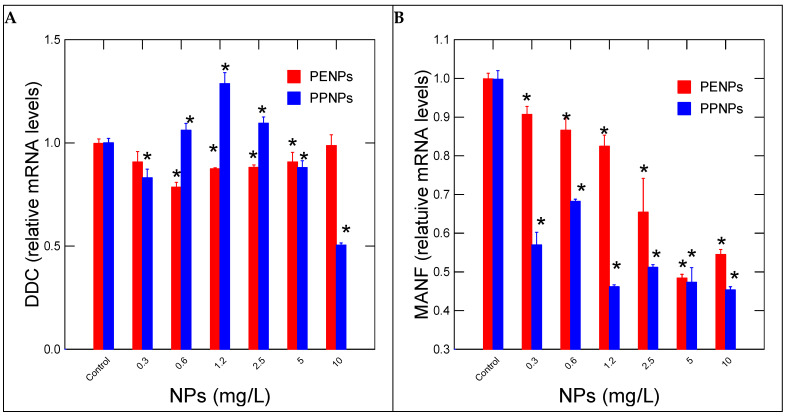
Dopaminergic activity in hydras exposed to plastic nanoparticles. Relative levels of DDC (**A**) and MANF (**B**) mRNA levels were determined in hydras exposed to NPs for 96h. The data represents the mean with the standard levels. The star (*) symbol represents significance at α < 0.05 levels from the control (vehicle only) and highlights the threshold concentrations.

Neural activity was followed in hydras by measuring changes in DDC and MANF gene expressions involved in dopamine decarboxylase (L-DOPA → dopamine) activity and cell repair of damaged dopaminergic neurons, respectively (Figure 3). For PeNPs, DDC gene expression was initially decreased at low-exposure concentrations and returned to control levels at 10 mg/L (Figure 3A). Correlation analysis revealed that DDC gene expression was correlated with CAT gene expression (r = 0.52). For PpNPs, the expression of DDC was significantly increased at concentrations up to 2.5 mg/L, followed by a significant decrease at 5 and 10 mg/L. Correlation analysis revealed that DDC gene expression was correlated with CAT (r = −0.4), SOD (r = 0.55), HPRT (r = 0.56) and OGG (r = 0.46). The levels of MANF involved in neural cell repair were also evaluated (Figure 3B). MANF gene expression was readily decreased for all exposure concentrations for both PE and PpNPs. For PeNPs, MANF gene expression was significantly correlated with SOD (r = 0.57), HPRT (r = 0.85) and OGG (r = 0.69). For PpNPs, gene expression of MANF was significantly correlated with SOD (r = 0.51), HPRT (r = 0.76) and OGG (r = 0.92). This suggests that cell repair activity of MANF involved oxidative stress and the oxidation of (8-oxoguanosine adduct formation).

**Figure 4:**
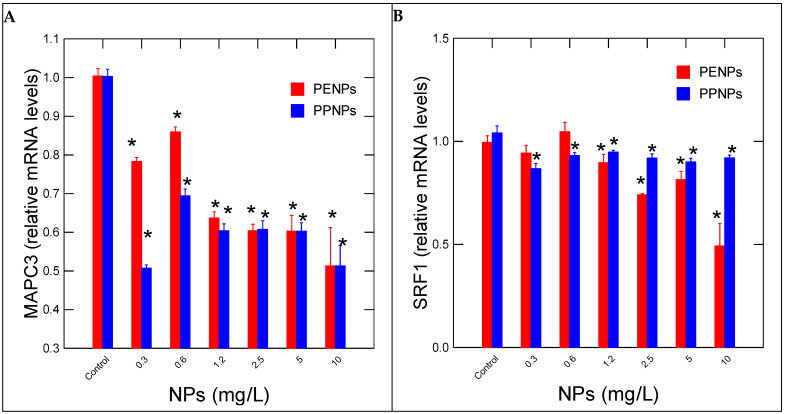
Ubiquitin signaling, autophagy and cell differentiation in hydra. Relative levels of MPAC3 (**A**) and SRF1 (**B**) mRNA levels were measured in hydras exposed to NPs for 96h. The data represents the mean with the standard levels. The star * symbol represents significance at α < 0.05 levels from the control (vehicle only) and highlights the threshold concentrations.

The expression of damaged protein salvage pathway (MAPC3) and differentiation (SRF1) in hydras exposed to PeNPs and PpNPs were examined (Figure 4). Both PeNPs and PpNPs were able to decrease gene expression for in MAPC3 at the lowest exposure concentration of 0.3 mg/L (Figure 4A). Correlation analysis revealed that MAPC3 mRNA levels were correlated with HPRT (r = 0.86), OGG (r = 0.86) and MANF (r = 0.79) for PeNPs. For PpNPs, MAPC3 levels were correlated with HPRT (r = 0.67), OGG (r = 0.88) and MANF (r = 0.9). Gene expression of SRF1 was decreased at 1.2 and 0.3 mg/L for PeNPs and PpNPs respectively (Figure 4B). The decrease was stronger for PpNPs compared to PeNPs. Correlation analysis revealed that SRF1 gene expression for PeNPs was significantly correlated with CAT (r = −0.43), SOD (r = 0.7), HPRT (r = 0.81), OGG (r = 0.6), MANF (r = 0.63) and MAPC3 (r = 0.76). For PpNPs, SRF1 was correlated with HPRT (r = 0.54), OGG (r = 0.67), MANF (r = 0.53) and MAPC3 (r = 0.72).

**Figure 5:**
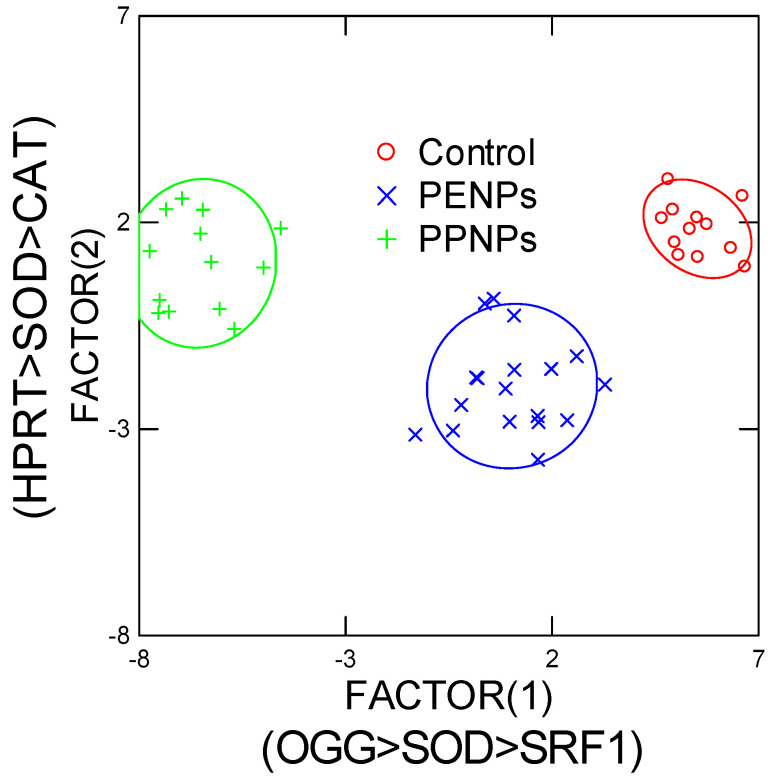
Discriminant function analysis of gene expression data in hydras exposed to nanoplastics. All the variance was explained with 100% predictive accuracy. The most important biomarkers are reported for each component (axis). The EC50 value for PpNs was 7 mg/L and produced more changes (farther from controls) than PeNPs, which displayed no morphological changes.

In the attempt to gain a global understanding of gene expression changes induced by PeNPs and PpNPs and to test the null hypothesis (i.e., the toxic effects of PENPS and PpNPs are identical), a discriminant function analysis was performed (Figure 5). The analysis revealed that the effects of each plastic’s nanoparticles were readily discriminated (100% efficiency) from each other based on DNA repair (OGG), SOD and SRF1 gene expressions (component 1 of x axis). HPRT and CAT gene expressions were also involved (y axis), but the discrimination was not clear on this axis. This suggests that oxidative stress (SOD involved in the production of H_2_O_2_ from oxygen radicals), reparation of oxidized DNA and cell differentiation were the main molecular events involved in the difference between PeNPs and PpNPs. It was noteworthy that PeNPs were closest to controls, suggesting less toxicity compared to PpNPs, in keeping with the observed stronger responses in gene expressions and the manifestation of sublethal morphological changes (EC50 of 7.1 mg/L).

## 4. Discussion

In this study, Pp- and PeNPs are both aliphatic polymers, spherical, same diameter (size) and uncoated. Hence, the observed effects are expected to be similar (null hypothesis), whereas different effects could arise from the manufacturing processes and the presence of various/variable additives such as phthalates and other plasticizers. Based on discriminant function analysis, the toxicity pattern of Pp and PeNPs differed based on oxidative stress biomarkers (SOD, OGG), especially for PpNPs, which displayed sublethal toxicity based on morphology. Moreover, PpNPs reduced the expression of SRF1 at the lowest concentration of 0.3 mg/L, which was observed for PeNPs only at higher concentrations of 2.5 mg/L (Figure 4). SRF1 is involved in tissue regeneration and stem cell factors essential for the development of the mesoderm and growth of tissues, including muscle/contractile cells [25,26]. These results further suggest including a more complete analysis of plastic nanoparticles (plasticizers and miscellaneous additives such flame retardants) in future studies. Plastic NPs were reported to induce oxidative stress in various organisms [15] ). Indeed, exposure to aged polyethylene terephthalate micro- and nanoplastic particles led to elevated reactive oxygen species formation in granulocytes and hyalinocytes (hemocytes) in *Mytilus edulis*. The particle concentration range was in the order of 10^1^ to 10^5^ particles/mL. In hydras, PENPs increased the expression of SOD at low concentrations (0.3–1.2 mg/L), followed by inhibitions at higher concentrations. CAT gene expression was only induced at the highest concentration of 10 mg/L. In another study with yeast, Pe teraphtalate nanoplastics caused oxidative stress and induced cell death [27]. PeNPs induced gene expression involved in oxidative stress (including Yap1, GPX1 and CTT1) and produced lipid peroxidation at concentrations between 2 and 5 mg/L. Evidence of stress at the levels of glucose deprivation, osmotic stress and heat shock proteins were also observed, based on the upregulation of MSN2 and MSN4 transcription factors. The study also revealed that autophagy had a protective role against PeNPs. This is consistent with the hydra responses, where MAPC3 gene expression, involved in protein denaturation salvage and autophagy, was downregulated by both Pe and PpNPs.

In respect to genotoxicity, gene expression in OGG was downregulated by both PeNPs and PpNPs, and the inhibition was stronger with the more toxic PpNPS, as with MAPC3 above. This suggests that decreased DNA repair activity is also associated with toxicity in hydras. In HepG2 cells exposed to various types of NPs, the toxicity of PetNPs and PVCNPs were more toxic than PSNPs of similar sizes. Toxicity was produced by oxidative stress and the inhibition of expression of DNA-repair genes in the p53 signaling pathway [28]. The reasons for the different toxicity between types of plastics are unclear, but it appears that the not the aliphatic nature of the plastic has a role in toxicity but the presence of plasticizers and other chemicals (stabilizers and polymerizers) used in these polymers. For example, in hydras, the toxicity of PpNPs was higher than PeNPs, based on morphological changes with an EC50 = 7 mg/L. The sublethal toxicity of 50 nm PsNPs was 3.6 mg/L [29], suggesting that PsNPs were somewhat more toxic than PpNPs of similar size. This suggests that not only the aliphatic properties of plastic NPs are associated with toxicity in hydras but other factors mentioned above. The toxicity of various plastic leachates to macrophage cell viability was investigated the cell viability and gene expression levels [30]. The size distribution of plastic nanoparticles leaching from the plastic materials was between 50 and 440 nm size, and the nanoparticles were irregular in shape. All types were able to reduce cell viability in a concentration dependent manner, and Pp, Ps and high-density Pe were more toxic than polyethylene methacrylate and polyethylene teraphtalate, based on cell viability. This suggests that the plastics made from more polar compounds (esters) are seemingly less toxic than less-polar plastic materials such as Pe, Ps and Pp NPs. It is noteworthy that polypropylene contamination was increased from the improper disposal of face masks during the COVID-19 pandemic.

In *Hyallela azteca* amphipods, 10-day exposure to either polyethylene or polypropylene microparticles resulted in decreased survival, with 4.64 × 10^4^ microplastics/mL and 71.43 microplastics/mL, respectively [31]. This corroborates the hydra toxicity data showing that PpNPs were more toxic than PeNPs. The study also showed that microplastic fibers were more toxic than particles and resided for longer periods of time in the digestive system of the amphipods. Although leachates from commercial plastics materials contain both nanoparticles and plasticizers, the toxicity of polyethylene, polystyrene and polypropylene leachates was investigated in *Dunaliella tertiolecta* algae [32]. Polypropylene and polystyrene reduced viability and growth at the lowest concentration of the leachate (3.1%). All leachates were able to induce oxidative stress in alga, but only Pp leachates produced genotoxic effects, in keeping with reduced DNA repair activity. Interestingly, GC-MS analysis revealed the absence of low molecular weight organic compounds usually found in plastics (plasticizers and flame retardants). This indicates that the polymeric materials (i.e., high molecular weight polymers and nanomaterials) and perhaps the associated metals/metalloids were responsible for toxicity.

The observed biphasic responses in DDC activity and decreased gene expression in MANF involved in the repair of dopaminergic neuronal cells suggest that these plastic nanoparticles are neurotoxic as well. DDC activity was increased at low exposure concentrations, suggesting that the hydras were in a wake and feeding state; conversely, decreased DDC gene expression at higher exposure concentration suggests that the hydras were in a sleep/resting state. Polyprolylene microplastics were shown to reduce acetylcholinesterase activity in Artemia salina shrimp, suggesting impacts on the nervous systems [33]. In another study with zebrafish, exposure to PSNPs (70 nm diameter) led to altered locomotor circadian rhythms in addition to oxidative stress [34]. This also led to disruptions in locomotor activity, aggressiveness, shoal formation and predator avoidance in fish exposed to 1.5 mg/L PSNPs. While dopamine, glutamate and histamine are associated with the awake phase, where feeding takes place, serotonin, melatonin and GABA neurotransmitters are involved in the sleep/resting phase of hydra [35,36] (Kanaya et al., 2020; Omond et al., 2022). The SRF1 gene is involved in the pacemaker/circadian cycles [37]. SRF1 is involved in the differentiation of I-cells into nerve cells, nemotocytes and gland cells. In adult hydras, the abundance of SRF transcript varies during the day, when its expression decreases 4h after feeding and is re-expressed after 12 h. The decrease in SRF1 by Pp (0.3 mg/L) and PeNPs (1.2 mg/L) suggests that the circadian rhythm of hydras is disrupted by those nanoplastics with stronger effects at lower concentrations, producing sublethal morphological changes. From the environmental protection perspective, the hydra assay is gaining more and more recognition as a sensitive invertebrate species and potential as an alternative method to fish testing [17]. This was supported by the present study showing sublethal effects at <0.3 mg/L at the gene-expression level. This suggests that plastic NPs could be harmful to hydras but not at the morphological level, especially in environments heavily polluted by plastics.

## 5. Conclusions

In conclusion, the exposure of hydras to PpNPs but not PeNPs led to sublethal morphological changes in the hydras. Both plastic materials were able to elicit changes in the expression of genes involved in oxidative stress, DNA repair activity, dopamine metabolisms and cell repair in dopaminergic neurons, protein–ubiquitin pathways (autophagy) and cell differentiation. The severity of the responses was greater with PpNPs, leading to sublethal toxicity compared to PeNPs and occurring at lower exposure concentrations. The following genes followed a biphasic response with increased gene expression at low concentrations, followed by a decrease at higher concentrations: SOD (PeNPs) and DDC (PpNPs). CAT expressions were repressed at low concentrations but was significantly increased at the highest concentration (10 mg/L) for both PeNPs and PpNPs. It is concluded that these plastic nanoparticles could produce oxidative stress, decreased DNA repair, protein salvaging and reduced tissue growth and development, leading to altered circadian rhythms at relatively high concentrations yet to be found in surface waters. However, this study sets the upper boundary of plastic nanoparticles that could pose a risk to hydras.

## Figures and Tables

**Table 1:** Gene expression target with the hydra.

Function	Gene Name	Forward/Reverse (5’---->3’)	Amplicon (bp)
Housekeeping genes	*60S acidic ribosomal protein P0-like* *Rplp0-1*	*CTG AGG CTG CTC TTC TTG CT/* *GGA CTG AAA ATG CTT CCG TTG T*	94
Housekeeping genes	*Elongation factor-1 alpha* *EL1*	*TGC TCC TGG ACA TCG TGA CT/* *CAA CGA TGA GTA CGG CAC AAT C*	77
*Autophagy and Ub-pathway*	*(Microtubule-associated protein 1 chain 3 light)* *MAPC3l*	*CCA GAG AAA GCG AGA ATC CGA/* *TGG AGA GCA TAC CAA CTG TCA T*	152
*Oxidative* *stress*	*Superoxide dismutase [Cu-Zn]-like* *SOD*	*ACC TGG TAA GCA CGG TTT TCA/* *TGC ACC ACT CCA TCT TTA CCA*	171
	*Catalase-like* *CAT*	*ACA GCC TCA ATG ACT GTT GGG/* *CCA CTC CAT TCA GAG CAG CC*	196
*DNA damage* *and repair*	*8-Oxoguanine DNA Glycosylase* *OGG*	*TGT GAC TGG AGT TGA AGA TGC T/* *ACT CCA GGC AAT GAG CAA AGA*	174
*Purine salvage* *pathway*	*Hypoxanthine-guanine phosphoribosyltransferase-like* *HPRT*	*GAA TTG AAC GCA TGG CTC GT/* *GTC TTG GCT GAA CCG AAA ACC*	98
*Regeneration and stem factor*	*Serum Response Factor* *SRF1*	*CTT GTG GCA TCG GAA ACA GG/* *TGC TTT GCC ACT TTC AGA GGT A*	84
*Neural activity*	*Dopa Decarboxylase* *DDC*	*GCC CCA GTT GAG CCA GAT AA/CAG TGA GTG ACA CCT GGC AT*	77
	*Mesencephalic astrocyte-derived neurotrophic factor homolog* (Reparation of damaged dopaminergic nerve cells) *MANF*	*CCA CTC GCA TAC TAC AAG CCT/* *ACA ACC ACT ACA AGT CTC ACC C*	180

**Table 2:** Correlation analysis. Significant (*p* < 0.05) correlations are highlighted in **bold**.

	Pe CAT	Pp CAT	Pe DDC	Pp DDC	Pe HPRT	Pp HPRT	Pe MANF	Pp MANF	Pe MAPC	Pp MAPC	Pe OGG	Pp OGG
PE CAT	1											
PP CAT	**0.82**	1										
PE DDC	**0.52**	**0.45**	1									
PP DDC	**−0.58**	**−0.45**	−0.33	1								
PE HPRT	**−0.45**	−0.13	−0.04	**0.46**	1							
PP HPRT	−0.23	−0.01	0.08	**0.53**	**0.90**	1						
PE MANF	−0.29	−0.12	−0.03	**0.50**	**0.86**	**0.94**	1					
PP MANF	0.11	0.24	0.30	0.17	**0.75**	**0.82**	**0.76**	1				
PE MAPC	−0.09	0.13	0.08	0.28	**0.87**	**0.85**	**0.80**	**0.92**	1			
PP MAPC	0.23	0.28	0.23	0.28	**0.57**	**0.69**	**0.59**	**0.90**	**0.84**	1		
PE OGG	−0.07	0.09	0.09	**0.34**	**0.72**	**0.78**	**0.68**	**0.81**	**0.86**	**0.81**	1	
PP OGG	−0.04	0.09	0.25	**0.47**	**0.77**	**0.89**	**0.81**	**0.93**	**0.87**	**0.89**	**0.82**	1
PE SOD	**−0.71**	**−0.46**	**−0.49**	**0.66**	**0.71**	**0.57**	**0.56**	0.16	**0.41**	0.07	**0.33**	0.31
PP SOD	**−0.42**	−0.18	−0.16	**0.53**	**0.81**	**0.88**	**0.93**	**0.56**	**0.63**	**0.36**	**0.55**	**0.64**
PES RF1	**−0.43**	−0.13	−0.29	**0.54**	**0.82**	**0.73**	**0.67**	**0.56**	**0.77**	**0.53**	**0.77**	**0.61**
PP SRF1	0.27	0.28	0.27	0.28	**0.32**	**0.54**	**0.43**	**0.63**	**0.58**	**0.72**	**0.56**	**0.67**
						**PE** **SOD**			**PP** **SOD**		**PE** **SRF1**	
PE CAT												
PP CAT												
PE DDC												
PP DDC												
PE HPRT												
PP HPRT												
PE MANF												
PP MANF												
PE MAPC												
PP MAPC												
PE OGG												
PP OGG												
PE SOD						1						
PP SOD						**0.68**			1			
PE SRF1						**0.74**			**0.64**		1	
PP SRF1						−0.12			0.25		**0.33**	

## Data Availability

The original contributions presented in this study are included in the article/Supplementary Material. Further inquiries can be directed to the corresponding author.

## Supplementary Materials

The following supporting information can be downloaded at https://www.mdpi.com/article/10.3390/nano15130954/s1: Figure S1. Morphological characteristics of Hydra vulgaris.
