# Supplementary material for: Sublethal Toxicity and Gene Expression Changes in Hydra vulgaris Exposed to Polyethylene and Polypropylene Nanoparticles
Source: Nanomaterials (Basel). 2025 Jun 20;15(13):954. doi: 10.3390/nano15130954 (PMC12250668; doi:10.3390/nano15130954)
Supplement: Supplementary file 1 [file nanomaterials-15-00954-s001.zip › nanomaterials-3676085-supplementary.pdf]

Supplementary material

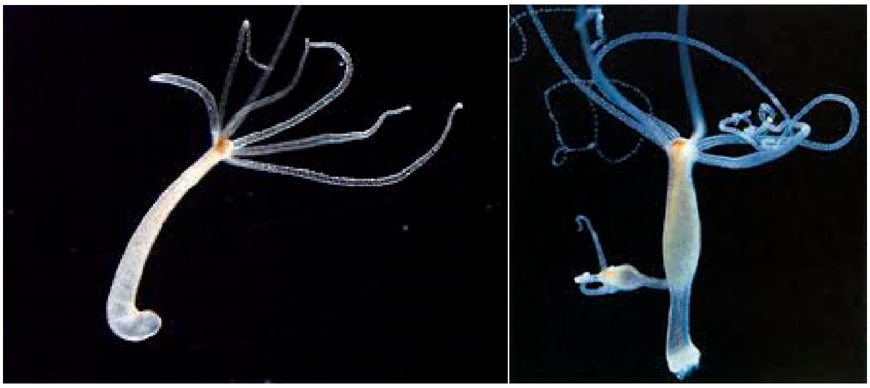

Figure S1. Morphological characteristics of Hydra vulgaris.

The head is composed of 5-7 tentacles to catch prey attached to the mouth and digestive system of the tubular body. The lower part of the body is reserved for reproduction where polyps emerge. The foot is attached to a solid substrate (glass here).
